# Advantages of a Novel Device for Arterial Catheter Securement in Anesthetized Dogs: A Pilot Randomized Clinical Trial

**DOI:** 10.3389/fvets.2019.00171

**Published:** 2019-06-04

**Authors:** Kazumasu Sasaki, Takuya Shiga, Ignacio Álvarez Gómez de Segura

**Affiliations:** ^1^Small Animal Emergency and Critical Care Service, Sendai Animal Care and Research Center, Sendai, Japan; ^2^Akita Cerebrospinal and Cardiovascular Center, Akita, Japan; ^3^Department of Preclinical Evaluation, Institute of Development, Aging and Cancer, Tohoku University, Sendai, Japan; ^4^Department of Anesthesiology and Perioperative Medicine, Tohoku University, Sendai, Japan; ^5^Department of Animal Medicine and Surgery, Faculty of Veterinary Medicine, University Complutense, Madrid, Spain

**Keywords:** arterial catheter, arterial catheter securement device, dog, dorsal pedal artery, intraoperative direct arterial blood pressure monitoring

## Abstract

Arterial catheters are used for intraoperative continuous direct blood pressure monitoring in dogs. Factors such as bending and occlusion of the cannula are believed to be involved in direct blood pressure measurement failure. However, no method has been proposed to improve the maintenance of arterial catheter patency in veterinary medicine. The aim of this pilot study was to evaluate the patency of arterial catheters when using an arterial catheter securement device in the dorsal pedal artery of dogs under general anesthesia. Client-owned dogs (*n* = 120) were anesthetized for surgical procedures, during which direct arterial blood pressure was monitored using an arterial catheter secured with conventional film dressing and medical tape. A securement device, allowing an angle of 12.5° to the skin surface of the dorsal pedal area, was used in 50% of the dogs (*n* = 60). Significant reductions were observed in the frequency of catheter flushing and rate of occlusion in the experimental group compared to the control group (13.3 vs. 35.0%, relative risk [RR]: 0.381, 95% confidence interval [CI]: 0.183–0.792, *P* = 0.001 and 8.3 vs. 23.3%, RR: 0.376, 95% CI: 0.145–0.977, *P* = 0.044, respectively). The Kaplan-Meier curves for assessing the probability of occlusion were significantly different between the groups (*P* = 0.042). In conclusion, this pilot study suggests that the novel arterial catheter securement device is effective for achieving stable securement of the catheter hub in the dorsal pedal artery and for maintaining a longer duration of arterial catheter patency in dogs under general anesthesia. Therefore, the use of an arterial catheter securement device in the dorsal pedal artery of dogs would be useful for continuous hemodynamic monitoring and improve patient safety when direct arterial blood pressure monitoring is required in dogs undergoing general anesthesia.

## Introduction

Blood pressure monitoring is routinely performed in veterinary patients under general anesthesia (1). Arterial catheters are used for intraoperative continuous direct blood pressure monitoring in the cases that are at risk of hemodynamic abnormalities (2). Insertion of an arterial catheter into the dorsal pedal artery in dogs has been found to be a safe procedure (3) and provides convenient access for multiple blood sampling and blood gas analysis (4). Hence, arterial catheterization is an important clinical technique when direct blood pressure measurement is required for hemodynamic monitoring in critically ill veterinary patients under general anesthesia.

Generally, the cannulation site is secured by commercially available film dressing and/or medical tape (4), and successful maintenance of arterial catheter patency is required for continuous direct arterial blood pressure monitoring. The requirement for frequent catheter flushing and incidence of indwelling arterial catheter occlusion are common occurrences in the clinical setting, which result in interference of the direct arterial blood pressure measurement. Therefore, reducing the frequency of catheter flushing and rate of occlusion may be the important steps for evaluating the hemodynamic status during the intraoperative period. Successful arterial catheter securement methods using specific film dressings (5) and arterial catheter securement devices (6) have been reported in human medicine. However, in veterinary medicine, despite the use of arterial catheters for direct arterial blood pressure monitoring, studies to determine the effects of catheter securement methods to enhance the arterial catheter patency have not been conducted.

Therefore, the aim of this pilot study was to investigate the arterial catheter patency with the use of a novel arterial catheter securement device in anesthetized dogs. We hypothesized that if the arterial catheter securement device achieved stability of the catheter hub in the dorsal pedal artery, the frequency of catheter flushing and incidence of occlusion would be reduced during direct arterial blood pressure monitoring in dogs under general anesthesia.

## Materials and Methods

### Study Design

This pilot study was a prospective, randomized, blinded study performed at the Small Animal Emergency and Critical Care Service, Sendai Animal Care and Research Center. The study was approved by the Institutional Ethical Committee on Animal Use of Akita Cerebrospinal and Cardiovascular Center (no. 18-02). The owners of the patients provided written informed client consent before enrolment for both participation in the study and for the publication of this report.

### Inclusion and Exclusion Criteria

**Figure 1:**
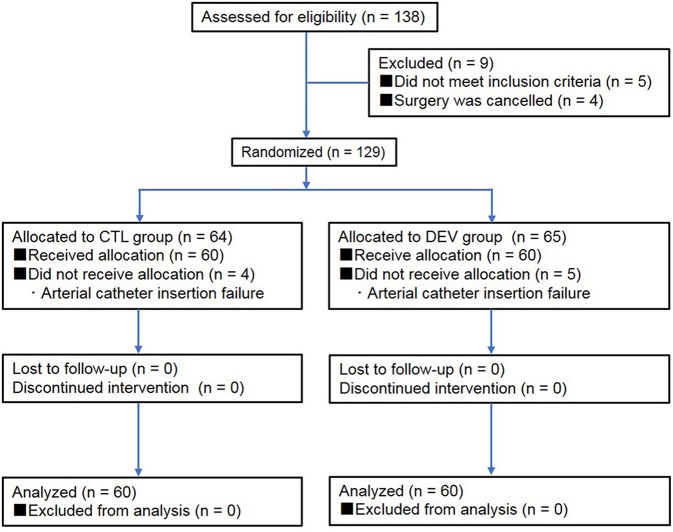
Flow chart of veterinary patient enrolment. CTL group: catheter securement with film dressing and medical tape. DEV group: catheter securement with the arterial catheter securement device, film dressing, and medical tape.

**Figure 2:**
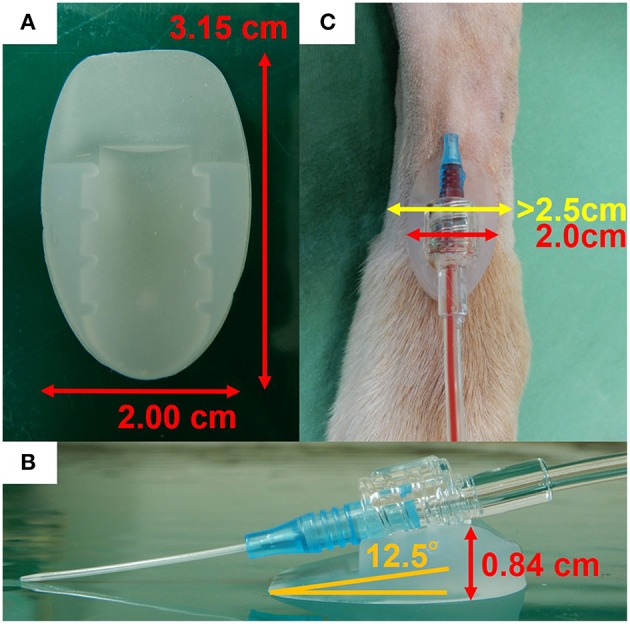
Positioning of the arterial catheter securement device. **(A)** Enlarged photograph of the arterial catheter securement device with size indications and **(B)** positioning of the catheter in the device cradle with an angle of 12.5° in relation to the surface. **(C)** Only dogs with a dorsal pedal area of >2.5 cm in width were studied.

**Figure 3:**
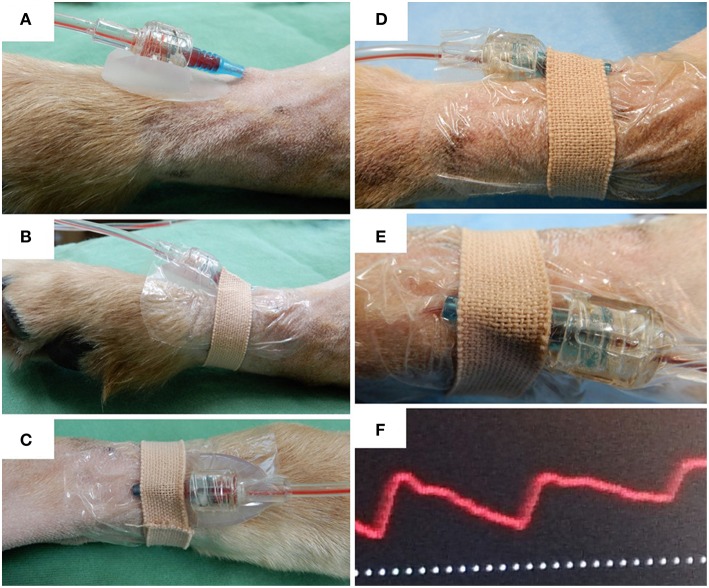
Arterial catheter securement in the dorsal pedal artery of dogs. **(A)** Relative positioning of the arterial catheter and the arterial catheter securement device. **(B)** Side view and **(C)** dorsal view of arterial catheter securement using the arterial catheter securement device, film dressing, and medical tape. **(D)** Side view of arterial catheter securement using film dressing and medical tape. **(E)** Bending of the arterial catheter secured with film dressing and medical tape (dorsal view). **(F)** Example of an abnormal pressure waveform observed in a dog with catheter occlusion.

The dogs were client-owned animals admitted to our veterinary hospital for the following surgical procedures: unilateral mastectomy, ovariohysterectomy (pyometra), cystolithectomy, or cholecystectomy. These dogs were assessed for eligibility in accordance with the Consolidated Standards of Reporting Trials (CONSORT) Guidelines (7) (Figure 1). Dogs with a dorsal pedal area of >2.5 cm in width (to accommodate the 2.0 cm width of the arterial catheter securement device) were included in the study (Figure 2C). The dogs were randomly assigned into one of the two groups as follows: the experimental (DEV) group (the arterial catheter was secured with the arterial catheter securement device, film dressing, and medical tape, Figures 3A–C) or the control (CTL) group (the arterial catheter was secured with film dressing and medical tape, Figures 3D,E) according to an automatically generated list using an online software, RESEARCH RANDOMIZER (8). The exclusion criteria were the presence of skin abnormalities in the dorsal pedal area, cancellation of the surgery, and arterial catheter insertion failure.

### Anesthesia and Intraoperative Monitoring

Anesthesia was induced via specific protocols corresponding to the animals' physical status and based on the preoperative physical examination findings, including clinical signs, echocardiography, radiography, complete blood cell count, coagulation system test, and serum biochemistry profile. Tracheal intubation was performed in all dogs, and mechanical ventilation was initiated using volume-controlled mechanical ventilator (Pro-Next +i/+s, ACOMA Medical Industry, Tokyo, Japan). The tidal volume (V_T_), respiratory rate (*f*_R_), positive end-expiratory pressure, and inspiratory pressure support were adjusted to maintain an end-tidal carbon dioxide (Pe'CO_2_) value of 35–45 mm Hg (4.7–6.0 kPa) under assist controlled or synchronized intermittent mandatory ventilation mode as appropriate in each animal. The animals underwent continuous monitoring of V_T_; heart rate; oxygen saturation of hemoglobin; *f*_R_; fraction of inspired oxygen; end-tidal sevoflurane, Pe'CO_2_; systolic, mean, and diastolic arterial pressure; and rectal temperature using a multiparameter monitor (Life Scope BSM-5192, Nihon Kohden, Tokyo, Japan) with an in-built automatic calibration system. The arterial catheter was inserted by a trained veterinary technician. Blood was sampled from the arterial catheter for blood gas analysis and laboratory tests during anesthesia.

### Arterial Catheter Securement Device

In this pilot study, we used an arterial catheter securement device with an insertion angle of 12.5° (A-namaran plus, Sun Arrow Kasei, Niigata, Japan), which is a protein-free cradle device made from silicone elastomer, 2.00 × 3.15 cm (width × length) in size, 0.84 cm in thickness, and 1.80 g in weight (Figures 2A,B). The interface configuration of the device can be fitted to the joint shape of the arterial catheter and pressure transducer.

### Arterial Catheter Insertion

In all dogs, the arterial catheter was inserted after the induction of general anesthesia. The hair was clipped over the proposed site and the skin was disinfected using 70% ethyl alcohol and chlorhexidine. A 22 gauge, 0.9 × 30 mm catheter (BD Insyte-A, Becton, Dickinson, UT, USA) was inserted into the dorsal pedal artery using a non-compliant pressure monitoring kit with a disposable transducer (DX-360, Nihon Kohden) connected to the multiparameter monitor as described above. The transducer was placed horizontally at the level of the right atrium as the zero reference point. The arterial catheter was flushed with 1 ml of heparinized saline (1) prepared by adding heparin (Heparin sodium, Mochida Pharmaceutical, Japan) to 0.9% saline for a final concentration of 4 IU/mL (3). Heparinized saline was infused at a rate of 3 mL/h with 300 mm Hg pressure applied to the infuser bags (9, 10). The arterial catheter was removed before endotracheal extubation.

### Clinical Evaluation

During the study period, all dogs were placed in dorsal recumbency. The arterial waveform was blindly monitored by a single investigator (KS). The number of required catheter flushing procedures, incidence of occlusion, and time to occlusion were also recorded by KS. In the present pilot study, catheter flushing with 1–2 ml was performed every time (maximum twice per catheter) the arterial waveform was considered to be unreliable in order to restore the arterial waveform to normal, which is composed of systolic upstroke, systolic peak pressure, systolic decline, dicrotic notch, and end-diastolic pressure (1, 2, 11, 12). An acceptable waveform was achieved if there was an appropriate curve response to the fast flush test (11). Failure to restore the arterial waveform and inability to draw blood, even after catheter flushing, were considered to indicate catheter occlusion (Figure 3F). The width of the dorsal pedal area, number of puncture attempts, catheter indwelling time, number of blood samplings, and number of times the dog was repositioned during surgery were recorded.

### Statistical Analyses

Data were tested for normality using Shapiro-Wilk test. The Mann-Whitney *U*-test was used to evaluate group differences in the patients' characteristics with regard to the width of the limb at the puncture site, number of puncture attempts, duration of indwelling arterial catheter, number of blood samplings, and number of times the dog was repositioned during surgery (Table 1). Fisher's exact test was used to determine the association between the variables with respect to the number of catheter flushing procedures and occlusion rate. Additionally, we estimated the relative risk (RR) of the frequency of catheter flushing and rate of occlusion. Kaplan-Meier curves were generated to assess the probability of occlusion, and comparisons between the groups were performed using a log-rank test. A *P*-value of 0.05 was considered statistically significant. Data are presented as the median (25–75 % interquartile range) unless otherwise stated. Statistical analyses were performed using GraphPad Prism version 6.0 (GraphPad Software Inc., La Jolla, CA, USA) and SigmaPlot version 13.0 (Systat Software Inc., San Jose, CA, USA).

**Table 1:** Characteristics of the dogs enrolled in the study.

	**CTL group (*n* = 60)**	**DEV group (*n* = 60)**	***P*-value**
Age (years)	7.5 (7.3–8.1)	7.5 (7.2–8.0)	0.876
Body weight (kg)	16.0 (13.2–19.0)	15.6 (13.1–18.4)	0.678
Width of the dorsal pedal area (cm)	2.8 (2.6–3.1)	3.0 (2.8–3.2)	0.083
Puncture attempts (n)	1 (1–2)	1 (1–2)	0.506
Catheter indwelling time (min)	176.0 (165.0–185.0)	170.5 (159.8–183.0)	0.171
Number of blood samplings (n)	0 (0–1)	0 (0–1)	0.705
Number of times the dogs were repositioned during surgery (n)	0 (0–1)	0 (0–1)	0.288

*Data are presented as the median (25–75% interquartile range). CTL group: catheter securement with film dressing and medical tape. DEV group: catheter securement with the arterial catheter securement device, film dressing, and medical tape*.

## Results

**Figure 4:**
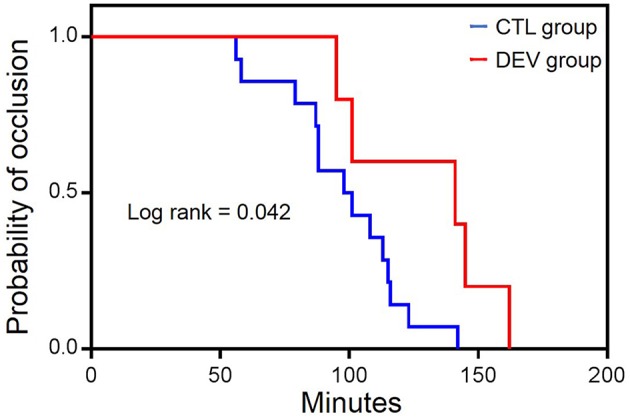
Kaplan-Meier survival curves of arterial catheter occlusion between groups. CTL group: catheter securement with film dressing and medical tape. DEV group: catheter securement with the arterial catheter securement device, film dressing, and medical tape.

Of a total of 138 adult dogs screened for eligibility, 129 were randomized and allocated to the CTL (*n* = 64) and DEV groups (*n* = 65). A total of 9 dogs were excluded from the randomization (5 did not meet the inclusion criteria and surgery was canceled for 4). Among the randomized dogs, 120 dogs (*n* = 60 per group, 97 female dogs and 23 male dogs) with a median weight of 15.6 kg (interquartile range: 13.1–18.7 kg) completed the trial, whereas 9 of these could not be analyzed (*n* = 4 in the CTL group and *n* = 5 in the DEV group). There were no significant differences in the patients' characteristics with regard to the width of the limb at the puncture site, number of puncture attempts, duration of arterial catheter insertion, number of blood samplings, and number of times the dog was repositioned during surgery (Table 1). During arterial catheter insertion into the dorsal pedal artery, the frequency of required catheter flushing in the DEV group (8/60, 13.3%) was significantly lower than that in the CTL group (21/60, 35.0%) (*P* = 0.001, relative risk [RR]: 0.381, 95% confidence interval [CI]: 0.183–0.792) (Table 2). In addition, the rate of catheter occlusion was lower in the DEV group (5/60, 8.3%) than in the CTL group (14/60, 23.3%) (*P* = 0.044, RR: 0.376, 95% CI: 0.145–0.977) (Table 2). The Kaplan-Meier curves assessing probability of occlusion were significantly different between the groups (hazard ratio: 0.404 [95 % CI: 0.141–0.857], *P* = 0.042) (Figure 4). The median (25–75% interquartile range) survival time of the catheter patency in the DEV and CTL groups was 141.0 min (101.0–145.0 min) and 99.5 min (87.3–114.5 min), respectively. No relevant postoperative complications such as contact dermatitis and tissue ischemia were observed in either group.

**Table 2:** Frequency of catheter flushing and rate of occlusion during arterial catheter placement.

	**CTL group** **(*n* = 60)**	**DEV group** **(*n* = 60)**	**Relative risk**	**95% Confidence interval**	***P*-value**
Catheter flushing	21/60 (35.0%)	8/60 (13.3%)	0.381	0.183–0.792	0.001
Occlusion	14/60 (23.3%)	5/60 (8.3%)	0.376	0.145–0.977	0.044

*CTL group: catheter securement with film dressing and medical tape. DEV group: catheter securement with the arterial catheter securement device, film dressing, and medical tape*.

## Discussion

We have demonstrated that the novel arterial catheter securement device allowed a significant decrease in the frequency of arterial catheter flushing and rate of occlusion. To the best of our knowledge, this is the first study to investigate the utility of an arterial catheter securement device in veterinary medicine. In the present pilot study, the characteristics of the dogs enrolled were not significantly different between groups, and we used the same film dressing and medical tape for all dogs. Therefore, our results were related to the use of the arterial catheter securement device only.

We could not compare our findings with those of previous related studies since no published data are available in this research field. However, a pilot study conducted at the Division of Anesthesiology, Niigata University Graduate School of Medicine and Dental Sciences in Japan indicated that the frequency of catheter flushing in the group using the experimental device (*n* = 49; 2/49, 4.1%) was lower than that in the control group (*n* = 48; 9/48, 18.8%) using conventional film dressings for arterial catheter securement in the radial artery during anesthesia (*P* < 0.05) (unpublished data). Since instability of the cannulation site is produced by contact of the skin surface with the joint segment of the arterial catheter and pressure transducer, the authors speculated that stable fixation was maintained by this device and thus enhanced the arterial catheter patency. Despite the fact that the arterial catheter securement device used in the present pilot study was developed for human medical purposes, the results of this pilot study demonstrate that the use of an arterial catheter securement device is clinically effective for promoting stable contact between the skin surface and the hub connection of the arterial catheter with a pressure transducer and is associated with a decreased frequency of catheter flushing and rate of occlusion in dogs. In addition, this securement device provided prolonged duration of catheter patency with an acceptable waveform during the study period. The study of the arterial catheter securement method has not been discussed in the current veterinary literature and thus, this investigation would be encouraging for future studies.

The following study limitations must be considered. First, this pilot study was conducted with a limited sample size (*n* = 60 per group) and in a single center; large scale multicenter studies are required to provide more powerful evidence for the efficacy of the arterial catheter securement device in dogs. Second, the device provides a catheter insertion angle of 12.5° for the human radial artery; thus, further study is warranted to assess whether a different insertion angle would yield better results in the dorsal pedal artery of dogs. Third, investigations using smaller breeds of dogs with narrower dorsal pedal areas than that in the present pilot study are required to confirm its universal efficacy and utility in dogs. Fourth, the arterial waveform was evaluated by an investigator with several years of experience in arterial waveform monitoring, but there were no objective methods to determine the normal morphology of the arterial waveform. Fifth, the effect of the type of catheter (diameter, material, and length) on the patency was not evaluated in this pilot study. Additional studies are needed to evaluate the effectiveness of the arterial catheter securement device in maintaining arterial catheter patency in different types of catheter. Sixth, our results could not be extended to the other arterial locations, including auricular artery, coccygeal artery, radial artery and femoral artery. Additional studies are needed to confirm the utility of the arterial catheter securement device in different arterial locations. Seventh, in the present pilot study, the duration of arterial catheter insertion into the dorsal pedal artery was < 3.5 h and further research is warranted to confirm if similar results can be achieved with longer periods of insertion time. Finally, we performed few blood samplings during the study period. In critically ill veterinary patients, a greater number of blood samplings than that in the present pilot study is required for blood gas analysis (4) and laboratory testing. Repetitive blood sampling may influence the frequency of flushing and incidence of occlusion. Further investigation is required to investigate the utility of the device in the veterinary critical care setting.

In conclusion, this pilot study provides preliminary results on the efficacy of the arterial catheter securement method when direct arterial blood pressure monitoring is required in dogs under general anesthesia. We believe that the novel arterial catheter securement device would be useful for continuous hemodynamic monitoring and enhance patient safety during general anesthesia. This clinical trial will contribute to the optimization of health care for dogs that require the insertion of invasive arterial catheters into the dorsal pedal artery, which has received little attention as a research topic.

## Data Availability

All datasets generated for this study are included in the manuscript.

## Ethics Statement

The study was approved by the Institutional Ethical Committee on Animal Use of Akita Cerebrospinal and Cardiovascular Center. The owners of patients provided written informed client consent before enrolment for both participation in the study and for the publication of this report.

## Author Contributions

KS: study design, data interpretation, statistical analysis, and preparation of the manuscript. TS: data interpretation and preparation of the manuscript. IAGS: literature research and preparation of the manuscript.

### Conflict of Interest Statement

The authors declare that the research was conducted in the absence of any commercial or financial relationships that could be construed as a potential conflict of interest.
